# Structural and Nonstructural Genes Contribute to the Genetic Diversity of RNA Viruses

**DOI:** 10.1128/mBio.01871-18

**Published:** 2018-10-30

**Authors:** Natalie D. Collins, Andrew S. Beck, Steven G. Widen, Thomas G. Wood, Stephen Higgs, Alan D. T. Barrett

**Affiliations:** a Department of Microbiology & Immunology, University of Texas Medical Branch, Galveston, Texas, USA; b Department of Pathology, University of Texas Medical Branch, Galveston, Texas, USA; c Sealy Institute for Vaccine Sciences, University of Texas Medical Branch, Galveston, Texas, USA; d Molecular Genomics Core Facility, University of Texas Medical Branch, Galveston, Texas, USA; e Department of Diagnostic Medicine/Pathobiology, College of Veterinary Medicine, Kansas State University, Manhattan, Kansas, USA; f Biosecurity Research Institute, Kansas State University, Manhattan, Kansas, USA; Baylor College of Medicine; Colorado State University; Curtin University

**Keywords:** RNA viruses, attenuation, diversity, viral population, vaccine, yellow fever

## Abstract

One paradigm to explain the complexity of viral RNA populations is that the low fidelity of the RNA-dependent RNA polymerase (RdRp) drives high mutation rates and consequently genetic diversity. Like most RNA viruses, wild-type yellow fever virus (YFV) replication is error-prone due to the lack of proofreading by the virus-encoded RdRp. However, there is evidence that replication of the live attenuated YF vaccine virus 17D, derived from wild-type strain Asibi, is less error-prone than wild-type RNA viruses. Recent studies comparing the genetic diversity of wild-type Asibi and 17D vaccine virus found that wild-type Asibi has the typical heterogeneous population of an RNA virus, while there is limited intra- and interpopulation variability of 17D vaccine virus. Utilizing chimeric and mutant infectious clone-derived viruses, we show that high and low genetic diversity profiles of wild-type Asibi virus and vaccine virus 17D, respectively, are multigenic. Introduction of either structural (pre-membrane and envelope) genes or NS2B or NS4B substitutions into the Asibi and 17D backbone resulted in altered variant population, nucleotide diversity, and mutation frequency compared to the parental viruses. Additionally, changes in genetic diversity of the chimeric and mutant viruses correlated with the phenotype of multiplication kinetics in human alveolar A549 cells. Overall, the paradigm that only the error-prone RdRp controls genetic diversity needs to be expanded to address the role of other genes in genetic diversity, and we hypothesize that it is the replication complex as a whole and not the RdRp alone that controls genetic diversity.

## Abstract (precis)

With the advent of advanced sequencing technology, studies of RNA viruses have shown that genetic diversity can contribute to both attenuation and virulence and the paradigm is that this is controlled by the error-prone RNA-dependent RNA polymerase (RdRp). Since wild-type yellow fever virus (YFV) strain Asibi has genetic diversity typical of a wild-type RNA virus, while 17D virus vaccine has limited diversity, it provides a unique opportunity to investigate RNA population theory in the context of a well-characterized live attenuated vaccine. Utilizing infectious clone-derived viruses, we show that genetic diversity of RNA viruses is complex and that multiple genes, including structural genes and NS2B and NS4B genes also contribute to genetic diversity. We suggest that the replication complex as a whole, rather than only RdRp, drives genetic diversity, at least for YFV.

## INTRODUCTION

Yellow fever virus (YFV) is a mosquito-borne virus that is the prototype member of the family *Flaviviridae*. The disease (YF) is controlled by live attenuated 17D vaccine strain, which was derived by 176 serial passages of wild-type Asibi virus in mouse and chicken tissue (1–3). Despite being developed in the 1930s, it is still considered to be one of the most effective and safe, live attenuated vaccines generated thus far. A single dose confers life-long immunity (4, 5).

The YFV genome is a single-stranded, positive-sense RNA molecule approximately 11,000 nucleotides in length and has a single open reading frame (ORF) that encodes three structural protein genes (capsid [C], pre-membrane/membrane [prM/M], and envelope [E]) and seven nonstructural (NS) protein genes in the gene order 5′-C-prM/M-E-NS1-NS2A-NS2B-NS3-NS4A-NS4B-NS5-3′ (6, 7). RNA viruses, such as YFV, exist as a population of genetically related viruses with varying genomic sequences that can contain both advantageous and nonadvantageous mutations. Studies with poliovirus have shown that viral population complexity contributes to the virulence and pathogenicity of the virus and allows the virus to overcome host barriers (8, 9). The rapid replication kinetics and the misincorporation of nucleotides by the low-fidelity RNA-dependent RNA polymerase (RdRp) of most RNA viruses contribute to high mutation rates and diversity of a viral population. The genetic diversity of wild-type Asibi virus and 17D-204 vaccine substrain has been compared, and wild-type Asibi virus possesses a highly diverse viral population typical of RNA viruses, while 17D vaccine strain is atypical and has limited genetic diversity (10, 11). This is consistent with an earlier study that proposed that 17D vaccine virus, unlike typical RNA viruses, has a high-fidelity RdRp with a theoretical error rate of 2 × 10^−7^ misincorporations per copied nucleotide (12). These results support the hypothesis that the limited viral population diversity of the 17D vaccine virus is a correlate of attenuation and contributes at least in part to the mechanism of attenuation.

Comparative genomic sequencing of wild-type Asibi strain and 17D vaccine showed that the vaccine differed from wild-type Asibi strain by 9 and 11 shared amino acid substitutions in the structural and NS protein genes, respectively (13, 14). The RdRp is encoded by NS5 and contains two (NS5-836 and NS5-900) of the amino acid substitutions. The RdRp is complexed with NS1, NS2A, NS2B, NS3, NS4A, and NS4B to form the virus replication complex (RC) (15, 16).

The flavivirus NS2B has not been as extensively studied as other NS proteins; it is known that the NS2B protein is a component of the RC and with NS3 forms the viral protease (17–19). The NS4B protein interacts directly with other components of the RC and is thought to be the scaffold for the RC (16, 20–25). Furthermore, it has been shown to antagonize the host innate immune response *in vitro* and in small animal models (26–29). Given the role of the NS2B and NS4B proteins in the RC, we hypothesized that the NS2B-L109I and NS4B-M95I substitutions that differentiate wild-type Asibi and 17D vaccine contribute to the limited viral population diversity of the 17D vaccine. This hypothesis was investigated using both structural and NS chimeric and mutant viruses to fully elucidate whether or not mutations outside the NS5 RdRp contribute to the diversity of a viral population. Our results suggest that, at the very least, the NS2B-L109I and NS4B-M95I substitutions in 17D vaccine contribute to limited genetic diversity. In addition, examination of structural chimeric (pre-membrane and envelope) viruses demonstrated the contribution of structural (pre-membrane and envelope) genes to viral population and genetic diversity. Overall, our studies indicate that mutations outside the RdRp contribute to genetic diversity and the limited diversity of the 17D vaccine is multigenic.

## RESULTS

### Generation and properties of infectious clone-derived viruses.

Infectious clone (IC)-derived viruses were recovered in two separate experiments, and their genomes were subjected to next generation sequencing (NGS); in total, 16 viruses were evaluated. IC-derived chimeric and mutant viruses were named based on the backbone (predominant viral template), followed by the mutation (e.g., 17D/Asibi NS4B-M95I denotes a 17D-204 vaccine virus backbone with a methionine [17D]-to-isoleucine [Asibi] substitution at position 95 in the NS4B protein) (see Fig. S1 in the supplemental material). The individual mutations correspond to the amino acid substitutions that differentiate between wild-type Asibi and 17D vaccine virus. The consensus sequences of IC-derived Asibi and Asibi backbone chimeric and mutant viruses were compared to wild-type Asibi (KF769016), while IC-derived 17D-204 virus and 17D backbone chimeric and mutant viruses were compared to IC-derived 17D-204 virus (KF769015). IC-derived 17D-204 and 17D-204 backbone chimeric and mutant viruses each had one additional consensus nucleotide change that was present in all four viruses at nucleotide 4025, which encoded NS2A-V173M substitution; the NS2A-V173M substitution in IC-derived 17D-204 virus was previously identified and did not affect the expected phenotype in Aedes aegypti mosquitoes (30); therefore, it is not likely to influence the results of this study. IC-derived Asibi and Asibi backbone chimeric and mutant viruses had three additional consensus nucleotide changes that were shared between the four viruses at nucleotides 304, 370, and 493, and all were noncoding. Asibi/17D prME had an additional consensus nucleotide change at nucleotide 2687, which encoded NS1-L80F substitution. The mean coverage of reads that spanned the genomes of the viruses used during this study ranged from approximately 900 to 8,000 (Table 1); therefore, virus reads were randomly down-sampled to the lowest mean coverage to correct for sampling depth bias. Multiple attempts to recover a virus for Asibi/17D prME with a mean coverage above 1,500 were unsuccessful, suggesting that interactions between structural (prM and E) and NS genes required for replication are affected by chimerization; the highest coverage achieved for Asibi/17D prME was 907. In terms of the recovery of infectious virus, chimerization and mutation of Asibi virus resulted in increased infectious virus yields, with IC-derived Asibi virus yielding approximately 10^4^ FFU/ml, while the Asibi backbone chimeric and mutant viruses yielded approximately 10^5^ to 10^6^ FFU/ml (Table 1). In comparison, IC-derived 17D-204 and 17D backbone chimeric and mutant viruses reached similar infectivity titers, approximately 10^6^ FFU/ml (Table 1). The focus sizes of IC-derived Asibi viruses were not uniform, measuring less than 1.5 mm, while the focus sizes of IC-derived 17D-204 virus were uniform and measured approximately 1 mm; in general, the focus sizes of Asibi and 17D backbone mutant viruses were consistent with their respective parental virus (Table 1). However, the foci of Asibi/17D prME measured less than 1 mm, while the foci of 17D/Asibi prME measured approximately 2 mm (Table 1).

**TABLE 1:** Descriptive statistics for the infectious clone (IC)-derived viruses

IC-derived virus	Mean coverage	Mean titer of recovered virus (FFU/ml)	Mean focus size
17D	4008	6.1 × 10^6^	Uniform; 1 mm
17D/Asibi prME	7988	4.1 × 10^6^	Uniform; 2 mm
17D/Asibi NS2B-L109I	2029	2.6 × 10^6^	Uniform; 1 mm
17D/Asibi NS4B-M95I	4364	4.7 × 10^6^	Uniform; 1 mm
Asibi/17D NS4B-I95M	4306	1.7 × 10^5^	Mixed; <1–1.5 mm
Asibi/17D NS2B-I109L	7160	2.2 × 10^6^	Mixed; <1–1.5 mm
Asibi/17D prME	793	4.3 × 10^5^	Uniform; <1 mm
Asibi	6891	6.0 × 10^4^	Mixed; <1–1.5 mm

10.1128/mBio.01871-18.1 FIG S1 Infectious clone constructs. a, b, and c are the Asibi backbone viruses, while c, d, and e are the 17D backbone viruses. a and d are the prME chimeric viruses, b and e are the NS2B-109 mutant virus, and c and f are the NS4B-95 mutant viruses. Red denotes Asibi genes, and blue denotes 17D-204 genes. Download FIG S1, EPS file, 2.4 MB. Copyright © 2018 Collins et al. 2018 Collins et al. This content is distributed under the terms of the Creative Commons Attribution 4.0 International license.

### IC-derived 17D-204 viruses are less diverse than IC-derived Asibi virus.

**FIG 1:**
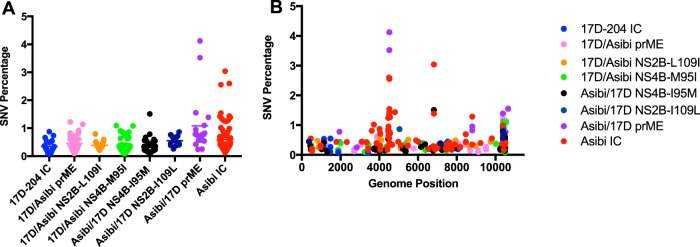
Structural and NS genes contribute to generation of diverse viral populations. (A) Total single nucleotide variant (SNV) percentages. (B) Total SNV percentages by genomic position. The IC-derived Asibi virus generates more variants and higher percentages of variants than IC-derived 17D-204 virus does. Introduction of 17D genes into the Asibi backbone decreased the number and frequency of variants identified compared to IC-derived Asibi virus. However, only introduction of NS4B-M95I mutation into the 17D backbone led to a noticeable increase in the number of variants identified compared to the IC-derived 17D-204 virus.

**FIG 2:**
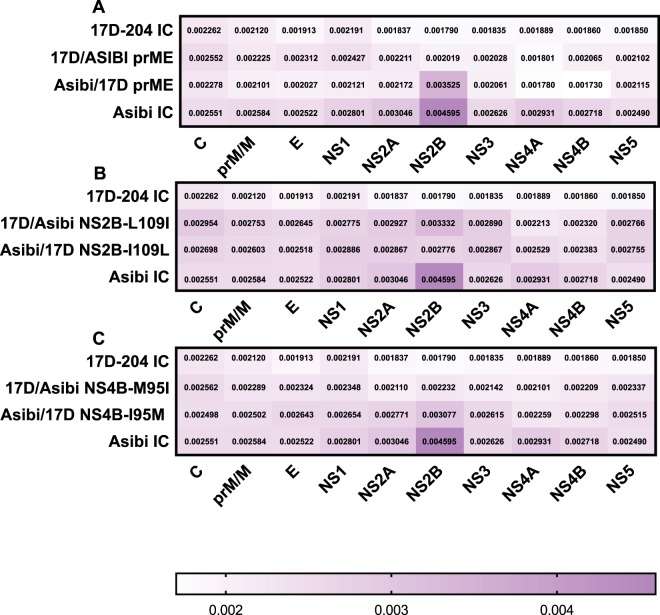
Introduction of structural and NS Asibi genes into the 17D backbone alters nucleotide diversity of IC-derived 17D-204 virus. (A) Structural chimeric viruses. (B and C) NS mutant viruses. The nucleotide diversity of IC-derived Asibi and 17D-204 viruses is statistically diverse in all genes except capsid and pre-membrane as determined by Mann-Whitney test. The nucleotide diversity of prME chimeric and NS2B-109 and NS4B-95 mutant viruses is statistically more diverse in multiple genes compared to IC-derived 17D-204 virus. The nucleotide diversity of prME chimeric virus was the only Asibi backbone virus that was statistically less diverse in multiple genes compared to IC-derived Asibi virus. Chimeric and mutant viruses were compared to parental IC-derived viruses using Kruskal-Wallis test, followed by Dunn’s comparison.

**FIG 3:**
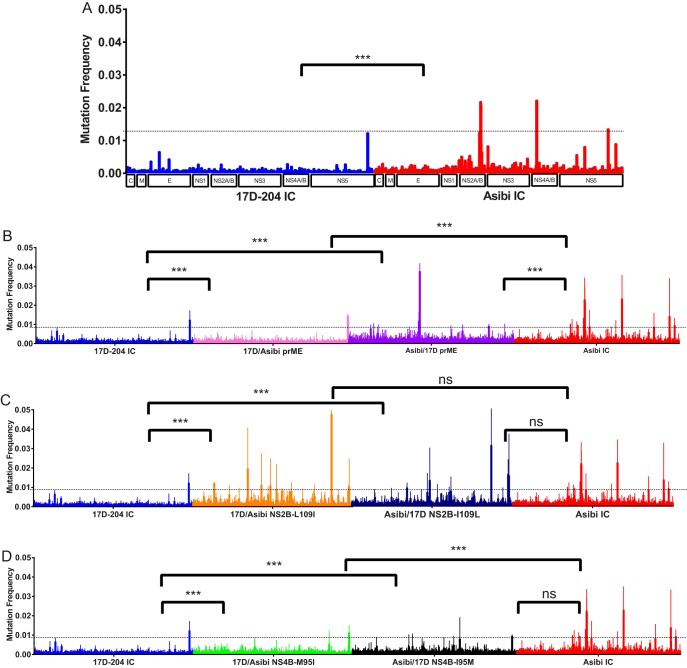
Introduction of structural and NS Asibi genes into the 17D backbone increased the mutation frequency of IC-derived 17D-204 virus. (A) Parental IC-derived Asibi and 17D-204 viruses. (B) Structural chimeric viruses. (C and D) NS mutant viruses. The mutation frequency of parental IC-derived viruses differed significantly (*P* value of <0.001), as determined by Mann-Whitney test. The mutation frequencies of all 17D backbone viruses were statistically significantly different compared to IC-derived 17D-204 virus (*P* values of < 0.001). In contrast, Asibi backbone viruses did not differ statistically compared to IC-derived Asibi. Chimeric and mutant viruses were compared to parental IC-derived virus using Kruskal-Wallis test, followed by Dunn’s comparison.

The Vphaser2 program (31) was used to identify trends in the genome sequences of the variant population that are characteristic of either IC-derived Asibi or IC-derived 17D-204 viruses to compare with the chimeric and mutant viruses. Variants that exceeded 1% of the viral population were further evaluated and classified as coding or noncoding mutations. A total of 78 variants were identified for IC-derived Asibi virus, including 9 positions in the ORF that exceeded 1% of the population; all were coding mutations. Positions 4505, 4517, and 6818 were present in both replicates; position 4505 encoded a NS2B-I109L amino acid substitution, which is a known amino acid substitution that differentiates wild-type Asibi from the 17D vaccine virus (Fig. 1 and Table 2). In comparison, 23 variants were identified for IC-derived 17D-204 virus, and none exceeded 1% (Fig. 1). These data were consistent with previous results by Beck and colleagues who demonstrated that the variant population of wild-type Asibi virus was more diverse than 17D-204 vaccine (10). Nucleotide diversity by gene was determined using Shannon’s entropy. Similar to the findings of Beck and colleagues (10), the nucleotide diversity in the envelope and NS genes of IC-derived Asibi virus were statistically more diverse than that of IC-derived 17D-204 virus; specifically, the nucleotide diversity of IC-derived Asibi virus was highest in the NS2B gene (Fig. 2 and Table S1). Furthermore, the mutation frequency of IC-derived Asibi virus was statistically higher than that of IC-derived 17D-204 virus; five positions within the coding region of IC-derived Asibi virus exceeded a mutation frequency of 0.01, while there was only one position in the ORF of IC-derived 17D virus that did so (Fig. 3). Collectively, the genetic diversity profile of IC-derived 17D-204 vaccine consisting of the generated variant population, nucleotide diversity, and mutation frequency, is distinctly different from that of IC-derived Asibi virus.

**TABLE 2:** Single nucleotide variants greater than 1% of the population for IC-derived Asibi virus

Reference base	Variant base	Genomic position	Gene region	Coding/noncoding	Variant frequency (%)
G	A	3724	NS2A	Yes; M1202I	1.02
G	A	4463	NS2B	Yes; D1449N	1.25
A	C	4505	NS2B	Yes; I1465L	2.60^*a*^
G	A	4517	NS2B	Yes; A1467T	2.56^*a*^
C	T	4520	NS2B	Yes; L1468F	1.42
A	C	4551	NS2B	Yes; H1478P	1.27
G	T	4808	NS3	Yes; A1564S	1.44
T	A	6818	NS4A	Yes; S2234T	3.04^*a*^
G	A	8801	NS5	Yes; E2895K	1.29

a The highest percentage is reported for SNV identified in both replicates.

10.1128/mBio.01871-18.2 TABLE S1 Nucleotide diversity comparison between IC-derived Asibi and 17D-204 viruses. Download Table S1, DOC file, 0.03 MB. Copyright © 2018 Collins et al. 2018 Collins et al. This content is distributed under the terms of the Creative Commons Attribution 4.0 International license.

### Structural protein genes contain determinants that contribute to diversity profile.

Studies on the mechanism of 17D attenuation have investigated the contributions of structural regions and NS4B gene on vector infectivity in *A. aegypti* and various combinations of the E protein domains in viral dissemination in the AG129 mouse model (30, 32–34). In this study, the relative contribution of structural protein genes to the lack of genetic diversity in the viral population of 17D vaccines was investigated using chimeric viruses with the prME structural protein genes “swapped”; note that viruses retained the capsid of the backbone virus, as there are no substitutions in the capsid protein between Asibi and 17D viruses. Nineteen variants were identified for Asibi/17D prME virus; four positions exceeded 1% of the population, one in the ORF was a coding mutation at position 4517 in the NS2B gene that was present in both replicates, and positions 10384, 10394, and 10652 were in the 3′ noncoding region (3′ NCR) (Fig. 1 and Table 3). Conversely, 32 variants were identified for 17D/Asibi prME virus, including positions 10384 and 10550 in the 3′ NCR that exceeded 1% (Fig. 1 and Table 3). It was noted that the nucleotide diversity of both Asibi/17D prME and 17D/Asibi prME viruses differed statistically from their respective backbone virus; the nucleotide diversity of Asibi/17D prME was statistically less diverse in all genes compared to IC-derived Asibi virus, while 17D/Asibi prME had two genes (E and NS5) that were statistically more diverse than IC-derived 17D-204 virus (Fig. 2 and Table S2). The mutation frequency of Asibi/17D prME virus was statistically lower compared to IC-derived Asibi virus, while the mutation frequency of 17D/Asibi prME virus was statistically higher compared to IC-derived 17D-204 (Fig. 3). Interestingly, positions 4463, 4505, and 4517 in the ORF of Asibi/17D prME that exceed a mutation frequency of 0.01 corresponded to the same positions as those for IC-derived Asibi virus. Additionally, genomic positions 4505 and 4517 correspond to variants greater than 1% of the population identified in IC-derived Asibi virus. Overall, the results of the prME chimeric viruses compared to their respective backbone viruses suggest that the structural (prM and E) genes contribute to the genetic diversity.

**TABLE 3:** Single nucleotide variant greater than 1% of the population for IC-derived chimeric and mutant viruses

Single nucleotide variant	Reference base	Variant base	Genomic position	Gene region	Coding/ noncoding	Variant frequency (%)
Asibi/17D prME						
	G	A	4517	NS2B	Yes; A1467T	4.12^*a*^
	C	T	10384	3′ NCR	No	1.39
	G	A	10394	3′ NCR	No	1.10
	G	T	10652	3′ NCR	No	1.55

17D/Asibi prME						
	C	T	10384	3′ NCR	No	1.22
	C	T	10550	3′ NCR	No	1.14

Asibi/17D NS4B-I95M						
	T	A	818	NS4A	Yes; S2234	1.50

17D/Asibi NS4B-M95I						
	C	T	10389	3′ NCR	No	1.08
	C	T	10550	3′ NCR	No	1.09

a The highest percentage is reported for SNV identified in both replicates.

10.1128/mBio.01871-18.3 TABLE S2 Nucleotide diversity comparison between prME chimeric viruses and parental IC-derived Asibi and 17D-204 viruses. Download Table S2, DOCX file, 0.01 MB. Copyright © 2018 Collins et al. 2018 Collins et al. This content is distributed under the terms of the Creative Commons Attribution 4.0 International license.

### NS protein genes outside the NS5 RdRp contribute to the viral population and genetic diversity of 17D vaccine.

Eleven of the 20 amino acids that differentiate wild-type Asibi and 17D vaccine viruses reside in the NS protein genes, including single amino acid substitutions in NS2B and NS4B. Therefore, as a proof of principle, Asibi/17D mutant viruses containing single substitutions at NS2B-109 or NS4B-95 were examined to investigate the potential role of NS genes other than the NS5 RdRp in genetic diversity.

Thirteen and 19 variants were identified for Asibi/17D NS2B-I109L and 17D/Asibi NS2B-L109I, respectively, none of which exceeded 1% of the population (Fig. 1). Asibi/17D NS2B-I109L displayed decreased nucleotide diversity in only the NS2B gene compared to IC-derived Asibi virus, while the nucleotide diversity of 17D/Asibi NS2B-L109I was increased in eight genes (C, E, NS1, NS2A, NS2B, NS3, NS4B, and NS5) compared to IC-derived 17D-204 virus (Fig. 2 and Table S3). The mutation frequency of Asibi/17D NS2B-I109L was not statistically different compared to IC-derived Asibi virus, while the mutation frequency of 17D/Asibi NS2B-L109I was statistically higher compared to that of IC-derived 17D-204 virus (Fig. 3). Additionally, seven positions in the coding region of the genome of 17D/Asibi NS2B-L109I exceeded a mutation frequency of 0.01.

10.1128/mBio.01871-18.4 TABLE S3 Nucleotide diversity comparison between NS2B-109 mutant viruses and parental IC-derived Asibi and 17D-204 viruses. Download Table S3, DOCX file, 0.01 MB. Copyright © 2018 Collins et al. 2018 Collins et al. This content is distributed under the terms of the Creative Commons Attribution 4.0 International license.

Forty-four variants were identified for both Asibi/17D NS4B-I95M and 17D/Asibi NS4B-M95I; one variant at genomic position 6818 for Asibi/17D NS4B-I95M exceeded 1% of the population and was a coding mutation, while two variants in the 3′ NCR (genomic positions 10389 and 10550) for 17D/Asibi NS4B-M95I exceeded 1% of the population (Fig. 1 and Table 3). The nucleotide diversity of Asibi/17D NS4B-I95M and 17D/Asibi NS4B-M95I varied compared to IC-derived Asibi and 17D-204 viruses. Nucleotide diversity in all genes (C, prM/M, E, NS1, NS2A, NS2B, NS3, NS4A, NS4B, and NS5) of Asibi/17D NS4B-I95M were consistent with the nucleotide diversity of IC-derived Asibi virus and differed statistically from IC-derived 17D-204 virus (Fig. 2 and Table S4). Unlike the other genes of Asibi/17D NS4B- I95M that differed statistically from IC-derived 17D-204 virus, the nucleotide diversity of the NS4A gene for Asibi/17D NS4B-I95M is similar to both IC-derived Asibi and 17D-204 viruses. The nucleotide diversity in the E, NS3, and NS5 genes of 17D/Asibi NS4B-M95I displayed increased diversity compared to IC-derived 17D-204; interestingly, the nucleotide diversity in the E, NS4B, and NS5 genes were indistinguishable from IC-derived Asibi virus (Fig. 2 and Table S4). The mutation frequency of Asibi/17D NS4B-I95M was not statistically different from that of IC-derived Asibi virus; however, there were no positions in the coding region of the genome of Asibi/17D NS4B-I95M that exceeded a mutation frequency of 0.01, while the mutation frequency of 17D/Asibi NS4B-M95I was statistically higher than that of IC-derived 17D-204 (Fig. 3).

10.1128/mBio.01871-18.5 TABLE S4 Nucleotide diversity comparison between NS4B-95 mutant viruses and parental IC-derived Asibi and 17D-204 viruses. Download Table S4, DOCX file, 0.01 MB. Copyright © 2018 Collins et al. 2018 Collins et al. This content is distributed under the terms of the Creative Commons Attribution 4.0 International license.

Overall, the altered genetic diversity profiles of the NS2B and NS4B mutant viruses suggests that mutations outside the NS5 RdRp contribute to the genetic diversity profile of wild-type Asibi and 17D vaccine virus.

### Phenotypic differences in multiplication kinetics of chimeric and mutant viruses.

**FIG 4:**
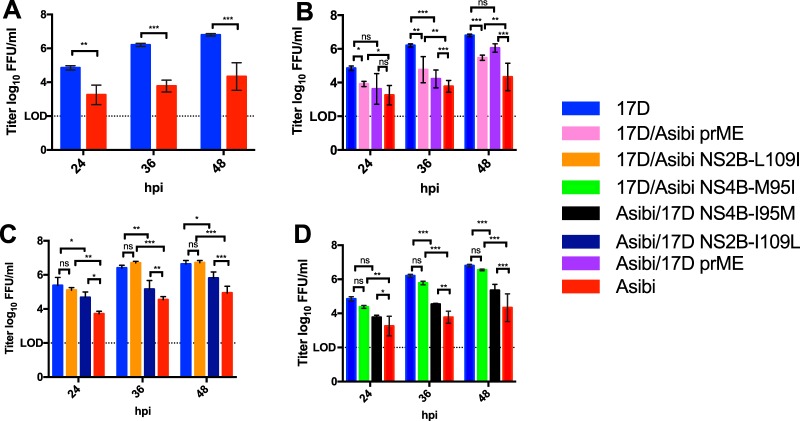
Multiplication kinetics of the IC at an MOI of 0.1 in A549 cells. (A) Parental IC-derived Asibi and 17D-204 viruses. (B) Structural chimeric viruses. (C and D) NS mutant viruses. Multiplication kinetics of IC-derived Asibi and 17D-204 viruses are statistically different at 24, 36, and 48 hpi as determined by a Student’s *t* test. The introduction of neither NS2B-L109 nor NS4B-M95I into the 17D-204 backbone altered the multiplication kinetics, while introduction of either NS2B-I109L or NS4B-I95M into the Asibi backbone did alter the multiplication kinetics. Chimeric and mutant viruses were compared to parental IC-derived virus using one-way ANOVA test, followed by Tukey’s multiple-comparison test. Statistical significance is indicated as follows: ns, not significant (*P* value = 0.12), *, *P* = 0.033; **, *P* = 0.002; ***, *P* < 0.001 (GraphPad Prism, version 7.0a).

To investigate whether or not the genetic diversity profile of the chimeric and mutant viruses translated to phenotypic differences, multiplication kinetics of parental IC-derived Asibi and 17D viruses were compared to the chimeric and mutant viruses in human alveolar A549 cells, which have a functional interferon-α/β receptor (Fig. 4).

Comparison of multiplication kinetics of wild-type IC-derived Asibi to IC-derived 17D-204 vaccine virus demonstrated that IC-derived 17D-204 virus multiplies more efficiently than IC- derived Asibi virus following infection of A549 cells at an MOI of 0.1, with significantly higher infectivity titers at 24 (*P* value = 0.007), 36 (*P* value < 0.001), and 48 (*P* value < 0.001) hours postinfection (hpi) (Fig. 4A); infectious virus was below the limit of detection at 12 hpi.

The infectivity titers of chimeric structural prME viruses displayed an intermediate phenotype compared to the two parental IC-derived viruses, such that the infectivity titers of both Asibi/17DprME and 17D/Asibi prME viruses were statistically different from their respective parental virus at 36 and 48 hpi (Fig. 4B). Importantly, the infectivity titers of Asibi/17D prME and 17D/Asibi prME viruses were statistically higher (*P* value = 0.001 and *P* value = 0.003, respectively) compared to those of IC-derived Asibi virus and statistically lower at 36 hpi (*P* value = 0.002 and *P* value < 0.001, respectively) compared to those of IC-derived 17D-204 virus.

The infectivity titers of NS2B-109 and NS4B-95 mutants were the most intriguing of the findings compared to backbone viruses (Fig. 4C and D). 17D/Asibi NS2B-L109I and 17D/Asibi NS4B-M95I multiplied just as efficiently as IC-derived 17D-204 virus did. However, the infectivity titers of Asibi/17D NS2B-I109L and Asibi/17D NS4B-I95M displayed an intermediate phenotype, differing from both IC-derived 17D virus and IC-derived Asibi virus at 36 and 48 hpi. Asibi/17D NS2B-I109L and Asibi/17D NS4B-I95M replicated to significantly higher titers at 36 hpi (*P* value of 0.004 and *P* value of 0.003, respectively) and 48 hpi (*P* value = 0.035 and *P* value < 0.001, respectively) compared to IC-derived Asibi virus. Conversely, Asibi/17D NS2B-I109L and Asibi/17D NS4B-I95M replicated to significantly lower titers at 36 hpi (*P* value = 0.004 and *P* value < 0.001, respectively) and 48 hpi (*P* value = 0.035 and *P* value < 0.001, respectively) compared to IC-derived 17D virus.

Taken together, the genetic diversity of prME chimeric viruses and NS2B-109 and NS4B-95 single-site mutant viruses translated to phenotypic differences in terms of multiplication kinetics of the viruses. However, the infectivity data for both 17D/Asibi NS2B-L109I and 17D/Asibi NS4B-M95I suggest that the contribution of both NS2B-L109I and NS4B-M95I amino acid substitutions to the genetic diversity of 17D vaccine are independent of multiplication kinetics.

## DISCUSSION

This study is the first to use IC-derived viruses to differentiate the genetic diversity profile of a live vaccine strain from the wild-type virus from which it was derived, as well as identifying structural and NS gene regions and mutations that contribute to genetic diversity. The results of this study suggest that the low genetic diversity profile of the 17D vaccine virus is due to multiple mutations, involving both structural (prM and E) and NS genes (at least NS2B and NS4B), and not exclusively the NS5 RdRp.

RNA viruses have high mutation rates, rapid multiplication kinetics/lysis time, possessing an error-prone RdRp due to lack of error-proofing replication, replication machinery, and elements within the terminal noncoding regions, all of which contribute to high mutation rates (8, 35, 36). Therefore, it was hypothesized that nucleotide misincorporation gives rise to subpopulations of genetically related viruses. To date, most studies on the genetic diversity of RNA viruses have focused on the role of the RdRp. The paradigm for viral population theory of RNA viruses is based on studies performed with poliovirus. Specifically, it was determined that the variant population of a high-fidelity mutant of poliovirus with a G64S amino acid substitution in the RdRp was less diverse than the variant population of wild-type poliovirus; coincidentally, the high-fidelity mutant was found to be less pathogenic than wild-type poliovirus (9, 37, 38). Conversely, mutations could also be introduced in the RdRp of poliovirus that would decrease replication fidelity and yield a RNA virus that was highly diverse, supporting the hypothesis that mutations in the RdRp of RNA viruses control mutation rate and genetic diversity (39). The contribution of mutations in the RdRp was further supported by studies utilizing other RNA viruses (40–46). Therefore, it has been generally accepted that the RdRp of RNA viruses was solely responsible for genetic diversity. However, the data in this paper support the hypothesis that mutations in the RC of RNA viruses as a whole, not just NS5, contribute to genetic diversity. Also, structural (prM and E) genes contribute to YFV genetic diversity, albeit probably by a different mechanism and likely associated with replication kinetics.

In this study, we show that, in general, introduction of 17D vaccine genes (prME, NS2B-109, and NS4B-95) into the wild-type Asibi virus backbone altered the genetic diversity of the chimeric and mutant viruses such that they differed compared to parental IC-derived Asibi virus. Not only were changes seen in the number and percentage of variants identified, but nucleotide diversity in some genes was not statistically different from IC-derived 17D-204 vaccine virus. Interestingly, introduction of Asibi genes (prME, NS2B-109, and NS4B-95) into the 17D backbone appeared to affect only nucleotide diversity and mutation frequency, and not the variant population. It was noted that both introduction of Asibi prME and NS4B-M95I genes into the 17D backbone accumulated two mutations in the 3′ NCR, while introduction of the Asibi NS2B-L109I gene did not. Thus, we cannot exclude the possibility that one or more of these nucleotide changes in the 3′ NCR could contribute to genetic diversity. However, taken together with the other results, they support the conclusion that the limited genetic diversity of the 17D vaccine is multigenic, where reversions in multiple genes would be necessary to return to a diversity profile consistent with wild-type Asibi virus. Support for this conclusion has been shown in vaccinees, where the attenuated phenotype was maintained along with minimal mutations of 17D vaccine in vaccinees (47). It has been suggested that the limited genetic diversity of 17D vaccine virus might in part explain the superior safety record for 17D vaccine (10, 48); indeed, the multiple attenuating mutations in both the structural (pre-membrane and envelope) and NS regions of the genome may maintain the limited diversity of the vaccine virus and consequently the attenuated phenotype. This warrants further evaluation.

Arthropod-borne viruses (arboviruses) occupy a unique position in terms of virus genome diversity, as they have a transmission cycle that requires two natural hosts, an arthropod and a vertebrate, that have very different selection pressures on restricting diversity/mutation rates. The necessity to replicate in two hosts allows for viral diversity and evolution; however, it also creates bottlenecks and subjects the virus to various selection pressures that influence viral diversity, including increased diversity in the arthropod host and decreased diversity in the vertebrate host, resulting in a lower rate of evolution of arboviruses than for other RNA viruses (49–53). Dudley and colleagues (54) showed that there was greater interhost variability observed in Zika virus-infected mosquitoes and nonhuman primates (NHPs) bitten by Zika virus-infected mosquitoes compared to the inoculum virus and NHPs that were inoculated with Zika virus by subcutaneous inoculation, suggesting that the virus encounters multiple bottlenecks during the transmission cycle. A study evaluating intrahost diversity of dengue 2 virus (DENV-2) found that greater than 90% of the viral population in DENV-2-infected humans were not observed in tissue samples of the mosquitoes allowed to feed on them (49). Additionally, the heterogeneity of viral populations within arthropod compartments has been documented (49, 51–53). The bottleneck encountered in arthropod vectors, coupled with the contribution of attenuating mutations in 17D vaccine virus that affect the ability to generate diverse populations during replication in mosquito and primate hosts, may contribute to the stability and safety of 17D vaccine.

To translate the genetic results to the virus phenotype, multiplication kinetics of the chimeric and mutant viruses was used as a phenotypic marker to further delineate the role of structural (pre-membrane and envelope) and NS genes in the genetic diversity profile of the 17D vaccine virus. As anticipated, introduction of either prME genes of 17D into the Asibi backbone or prME genes of Asibi into the 17D backbone altered the multiplication kinetics of the viruses compared to their respective parental viruses. The NS2B-109 and NS4B-95 amino acid substitutions had differing effects depending on the backbone virus; the NS2B-109 and NS4B-95 substitutions did not alter the multiplication kinetics of IC-derived 17D virus, while they did alter the multiplication kinetics of IC-derived Asibi virus. In comparison, previous studies showed that differences in the multiplication kinetics of poliovirus and Chikungunya virus (CHIKV) fidelity mutants were not observed (9, 37, 39, 41); however, interferon-incompetent cell lines were used in those studies. Similarly, C6/36 mosquito cells are often used to study flaviviruses, but these cells have a defective innate immune response comparable to the interferon response defect in Vero (vertebrate) cells. Conversely, studies with WNV fidelity mutants demonstrated that mutations that altered viral diversity, nearly abolishing vector infectivity in both colonized and field populations of *Culex quinquefasciatus* (43). Thus, determination of phenotypic differences of the chimeric and mutant viruses in this study was likely attributable at least in part to the utilization of an interferon-competent cell line. Additionally, the multiple attenuating mutations present in the genome of 17D vaccine contribute to the maintenance of the 17D phenotype of 17D/Asibi NS2B-L109I and 17D/Asibi NS4B-M95I mutant viruses. This conclusion is supported by previous studies with the NS4B mutant viruses in *A. aegypti* mosquitoes where dissemination was significantly decreased for the Asibi backbone viruses compared to IC-derived Asibi virus, while the 17D backbone viruses were consistent with IC-derived 17D virus (30). Furthermore, the prME chimeric mutants in this study demonstrate that the alterations in the genetic diversity profile of structural (pre-membrane and envelope) genes are unrelated to replication fidelity.

Overall, the phenotypes obtained from studies of multiplication kinetics support the genetic analysis of the viruses, namely, substitutions in the structural (pre-membrane and envelope), NS2B, and NS4B protein genes of Asibi and 17D viruses that alter genetic diversity also correlate with phenotypic changes. Additionally, it is speculated that the contribution of the NS2B-109 and NS4B-95 mutations to the genetic diversity profile is likely associated with their roles in the RC (16–25). This study showed that mutations in the RC, outside the NS5 gene that encodes the RdRp, contribute to genetic diversity and phenotypic changes; thus, the limited diversity of the 17D vaccine could contribute to the mechanism of attenuation. The genetic profile of multigenic mutations in the 17D vaccine renders the vaccine unable to generate a diverse viral population to combat host selection pressures, resulting in attenuation of virulence. Thus, as we suggested previously, lack of genetic diversity may be a biomarker for the development of live attenuated vaccine viruses (10).

## MATERIALS AND METHODS

### Preparation of YFV infectious clones.

YF infectious clones (IC) were generated by site-directed mutagenesis (SDM) of either pANCR 17D (IC) or pANCR Asibi IC as a template. A QuikChange XLII SDM kit (Stratagene) and mutagenic primer sets were used to generate infectious clones per the manufacturer’s protocol. Stock cDNA YF IC were amplified in Escherichia coli (MC1061), and plasmid was harvested by large-scale extraction. Briefly, 200-ml cultures were incubated at 37°C and 200 rpm for 12 to 14 h. The culture was then pelleted at 4°C by centrifugation and resuspended in cold glucose-Tris-EDTA. A 0.2 N NaOH–1% SDS solution was used for cell lysis, followed by 3 M KOAc (pH 4.8) to neutralize the reaction. The plasmid was pelleted by isopropanol precipitation and then resuspended in TE buffer, followed by treatment with RNase A for 30 min at 37°C, and purification by phenol-chloroform extraction. The purified plasmid was further concentrated using Clean and Concentrate kit according to the manufacturer’s instructions (Zymo).

cDNA YF ICs (4 μl/ml) were linearized by digestion with XhoI for 17D backbone viruses and NruI for Asibi backbone viruses at 37°C for 2 h. Linearized cDNA was further digested with 50 μl/ml proteinase K, purified twice by phenol-chloroform and once by chloroform extraction, followed by ethanol precipitation. Full-length YF RNA was generated by *in vitro* transcription of purified cDNA using SP6-Scribe standard RNA *in vitro* transcription kit (Cellscript) according to the manufacturer’s protocol; the reaction mixture was incubated at 37°C for 2.5 h. The entire reaction mixture was electroporated into 6.7 × 10^6^ monkey kidney Vero cells in a prechilled 2-mm cuvette using a Bio-Rad Gene Pulser set at 1.5 kV, infinite ohms, and 25 µF. Electroporated cells were allowed to recover for 10 min at room temperature and then transferred into 15 ml of prewarmed MEM supplemented with 8% fetal bovine serum (FBS), L-glutamine, sodium bicarbonate, and penicillin-streptomycin in a T-75 culture flask. Cell supernatant was collected when infected cells showed 80% cytopathic effect (CPE). RNA from the YF ICs were extracted using QIAamp Viral RNA minikit (Qiagen), following the manufacturer’s guidelines.

### Data analysis of NGS for YFV infectious clones.

RNA was extracted from IC-derived viruses, and NGS libraries were constructed using TruSeq RNA v2 kit (Illumina) as recommended by the manufacturer and then sequenced on an Illumina HiSeq1500 instrument. Paired-end reads were quality trimmed to minimum 35 bases and a minimum quality score of 35 using trimmomatic (version 0.22) and then realigned to a reference genome for wild-type Asibi virus (GenBank accession no. AY640589) using Bowtie2 local alignment mode and very sensitive setting (version 2.2.4). PCR duplicates were removed using Picard-tools (version 1.120) using default settings and an optical duplicate distance of 0. Qualimap (version 2.2) was used to assess the quality and determine the mean read coverage of NGS data sets. The NGS data sets were matched by random down-sampling using Picard-tools (version 1.120) to the virus with the lowest mean coverage. Vphaser2 (version 2.0) was used to determine variant population; all single nucleotide variants controlled for false discovery and strand bias were considered in the analysis (31). Sequence data that support findings of this study have been deposited in ArrayExpress accession no. E-MTAB-6614 (https://www.ebi.ac.uk/arrayexpress/experiments/E-MTAB-6614).

Diversity indices utilizing the NGS data sets were determined for the ORF by using deepSNV (version 1.16.0) and local R scripts (version 3.2.4). Briefly, nucleotide counts for genomic positions were determined, and then mutation frequencies were calculated for each genomic position; the total number of mutant bases called for a genomic position was divided by the total number of bases called at the genomic position as previously described (55). Nucleotide counts were then converted to relative nucleotide frequencies. Nucleotide diversity by gene regions was determined by calculating Shannon’s entropy using the nucleotide frequencies as previously described (56). Kruskal-Wallis test and Dunn’s multiple-comparison test were used to assess the significance of diversity index results; unless otherwise stated, statistical significance is indicated as shown in the parentheses: *P* value = 0.12 (ns [not significant]), *P* = 0.033 (*), 0.002 (**), and *P* < 0.001 (***) (GraphPad Prism, version 7.0a).

### Infection of human alveolar A549 cells.

A549 cells were purchased from ATCC and maintained according to ATCC guidelines in minimal essential medium (MEM) supplemented with 8% fetal bovine serum, L-glutamine, sodium bicarbonate, and penicillin-streptomycin. Prior to infection, A549 cells were allowed to adhere to plates at 37°C under 5% CO_2_ for at least 18 h. Adherent cells were infected at an MOI of 0.1 in triplicate and incubated at room temperature for 30 min. After the inoculum was removed, cells were washed 3 times with PBS and then incubated with 2 ml of MEM supplemented with 2% FBS, L-glutamine, sodium bicarbonate, and penicillin-streptomycin at 37°C under 5% CO_2_; cell supernatant collected at 0, 12, 24, 36, and 48 hpi. Cell supernatant was stored at −80°C until assayed. Infectious virus was titrated in duplicate by immunohistochemical focus-forming assay (FFA) in Vero cells. FFAs were fixed with 1 part acetone to 1 part methanol at 4 dpi and stained using YFV Asibi mouse immune ascitic fluid polyclonal antibody as the primary antibody. Multiplication kinetics of IC-derived Asibi and 17D-204 viruses were first compared using Student’s *t* test, before comparing the IC-derived chimeric and mutant viruses using one-way ANOVA and Tukey’s multiple-comparison test; *P* value = 0.12 (ns), *P =* 0.033 (*), *P =* 0.002 (**), and *P* < 0.001 (***) (GraphPad Prism, version 7.0a).
