# Supplementary material for: Structural and Nonstructural Genes Contribute to the Genetic Diversity of RNA Viruses
Source: mBio. 2018 Oct 30;9(5):e01871-18. doi: 10.1128/mBio.01871-18 (PMC6212827; doi:10.1128/mBio.01871-18)
Supplement: TABLE S1 [file mbo005184108st1.doc]

| **Supplemental Table 1. Nucleotide diversity comparison between IC-derived Asibi and 17D-204 Viruses.** | | | | | | | | | | |
| --- | --- | --- | --- | --- | --- | --- | --- | --- | --- | --- |
|  | C | prM | E | NS1 | NS2A | NS2B | NS3 | NS4A | NS4B | NS5 |
| Asibi IC vs 17D-204 IC | ns | ns | *** | *** | *** | *** | *** | *** | *** | *** |
|  | | | | | | | | | | |

P-value = .12 (ns), 0.033 (*), 0.002 (**), and < 0.001 (***).
