# Supplementary material for: Structural and Nonstructural Genes Contribute to the Genetic Diversity of RNA Viruses
Source: mBio. 2018 Oct 30;9(5):e01871-18. doi: 10.1128/mBio.01871-18 (PMC6212827; doi:10.1128/mBio.01871-18)
Supplement: TABLE S3 [file mbo005184108st3.docx]

| **Supplemental Table 3. Nucleotide diversity comparison between NS2B-109 mutant viruses and parental IC-derived Asibi and 17D-204 viruses.** | | | | | | | | | | | |
| --- | --- | --- | --- | --- | --- | --- | --- | --- | --- | --- | --- |
|  |  | C | prM | E | NS1 | NS2A | NS2B | NS3 | NS4A | NS4B | NS5 |
| 17D/Asibi NS2B-I109L | 17D IC | * | ns | *** | *** | *** | *** | *** | ns | ** | *** |
|  | Asibi IC | ns | ns | ns | ns | ns | ns | ns | * | ns | * |
| Asibi/17D NS2B- L109I | 17D IC | ns | ns | *** | *** | *** | ** | *** | ns | ** | *** |
|  | Asibi IC | ns | ns | ns | ns | ns | ** | ns | ns | ns | ns |
